# Fallopian Tube's Placental Site Nodule: A Case Report

**DOI:** 10.1155/crip/4514707

**Published:** 2025-09-04

**Authors:** Nicolas Chauveau, Jean-Christophe Tille, Jessica Kartotaroeno

**Affiliations:** ^1^Diagnostic Department, Division of Clinical Pathology, University Hospital of Geneva, Geneva, Switzerland; ^2^Division of Anatomy Cytology Pathology and Forensic Medicine, Raymond-Poincaré Hospital APHP, Garches, France

## Abstract

**Introduction:** Placental site nodules (PSNs) are uncommon lesions typically found in uterine specimens and extremely rarely in extrauterine locations such as the fallopian tubes. PSNs are usually discovered incidentally and result from prior implantation sites. This case report describes an unexpected PSN found in a fallopian tube during a cesarean section with concurrent tubal sterilization.

**Case Presentation:** A 38-year-old multiparous woman (gravida 4, para 2) with a previous vaginal delivery underwent tubal sterilization during a cesarean section. The surgical procedure was uneventful, with no macroscopic abnormalities noted. The patient's medical history included treatment for an ectopic pregnancy with methotrexate. Pathological examination revealed the right fallopian tube to be unremarkable. However, the left fallopian tube contained a 0.3 cm nodule within its wall, characterized by central hyalinization, dystrophic calcifications, and peripheral intermediate trophoblast cells. Immunohistochemical analysis demonstrated GATA3 positivity and a low proliferative index (MIB-1). The absence of mitotic activity, necrosis, and typical morphology confirmed the diagnosis of a PSN.

**Discussion:** PSNs are benign lesions derived from intermediate extravillous trophoblasts. Their identification relies on both morphological characteristics and immunohistochemical staining. The differential diagnosis includes various trophoblastic diseases, which can be distinguished from PSNs by their specific features. This case contributes to the limited literature on extrauterine PSNs, highlighting the importance of recognizing these lesions in atypical locations and differentiating them from pathologies that are more aggressive.

**Conclusion:** This case highlights the rarity of PSNs in the fallopian tubes and underscores the importance of comprehensive pathological analysis for an accurate diagnosis.

## 1. Introduction

A placental site nodule (PSN) is an uncommon lesion primarily described in hysterectomy [1] specimens, sometimes by hysteroscopy [2], and is extremely rare in extrauterine locations such as the fallopian tubes, ovaries, or paratubal tissues and ligaments [3–6]. This lesion is most often discovered incidentally and represents the sequelae of old implantation sites. In this case, we report the fortuitous discovery of a PSN localized in the fallopian tube.

## 2. Case Presentation

A 38-year-old multiparous woman (gravida 4, para 2) who had previously delivered vaginally underwent tubal sterilization during a cesarean section. The surgical procedure was uneventful, and no macroscopic abnormalities were observed. The patient's medical history included a left ectopic pregnancy treated with two doses of methotrexate 4 years prior.

**Figure 1:**
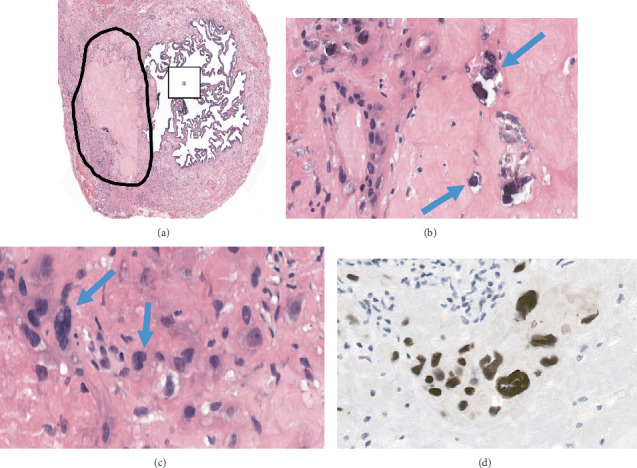
(a) Tranversation section for the fallopian tube, with the lesion in the submucosae and in the muscular wall. Circle = lesion, ∗ = lumen of the fallopian tube. HE staining, magnification 40x. (b) The lesion is composed of a hyalin stroma and dystrophic calcifications (arrows). HE staining, magnification 400x. (c) At the periphery of the lesion, there is a large cell with eosinophilic cytoplasm and large hyperchromatic nuclei corresponding to intermediate trophoblast cells (arrows). HE staining, magnification 400x. (d) GATA3 immunostaining was positive in these cells with a brown staining of the nucleus, confirming that they are trophoblastic cells, magnification 400x.

Pathological examination of the fallopian tubes showed no macroscopic lesions. Histology of the right fallopian tube revealed a preserved lumen with no abnormalities (Figure 1a). However, the left fallopian tube contained a 0.3 cm nodule within its wall. The nodule was characterized by central hyalinization, acellularity, and dystrophic calcifications (Figure 1b). At the periphery, intermediate trophoblast cells with eosinophilic cytoplasm, large hyperchromatic nuclei (Figure 1c), and occasional multinucleation were observed. There was no evidence of mitotic activity or necrosis.

Immunohistochemical staining revealed GATA3 positivity in the trophoblast cells (Figure 1d) and a low proliferative index (MIB-1 at 3%). The findings were consistent with a diagnosis of a PSN without CD146 positivity [7].

## 3. Discussion

Intermediate trophoblast lesions in the fallopian tube are exceptionally rare. PSNs, the most common of these lesions, originate from intermediate extravillous trophoblasts of chorionic type. This entity has been known and described since 1988 [8], but cases with extrauterine localization remain anecdotal and are always discovered incidentally. Recognition of intermediate trophoblast lesions is primarily morphological and, if necessary, aided by immunohistochemistry. Typical PSNs are well circumscribed, often surrounded by a mild chronic inflammatory infiltrate, and sometimes accompanied by decidualized stroma. The nodules are composed of scattered cells, arranged in cords or small clusters, with hyperchromatic nuclei of variable size. The cytoplasm may be eosinophilic, vacuolated, and rich in glycogen. Centrally, the nodule is hyalinized with few mitotic figures. Conventional immunostaining, such as AE1/AE3 cytokeratins, highlights the intermediate trophoblast cells [9].

A PSN is benign, and the main differential diagnosis includes trophoblastic diseases [10] such as epithelioid trophoblastic tumors (ETTs) [11], placental site trophoblastic tumors (PSTTs) [12], atypical PSNs, or, in certain locations, squamous cell carcinoma of the cervix [13].

The distinction between a malignant and benign lesion is of paramount importance for further management and prognosis. In the case of ETT or PSTT, these lesions are less sensitive to chemotherapy than choriocarcinoma, and surgical treatment of the lesions is the first choice after ruling out possible distant metastases (pulmonary, hepatic, or cerebral) [14]. In advanced cases, polychemotherapy is the standard treatment.

In all cases, histopathological cell morphology, mitotic activity, and immunohistochemistry such as the proliferative index (Ki67), p63, CD146, and human placental lactogen (hPL) [15] should guide the pathologist toward the correct diagnosis.

For tubal localization specifically, ETTs, though not described in this context, would likely present similarly to uterine cases. Such lesions are generally larger than a PSN and present distinct histopathological features, including more atypical cells with round nuclei, small nucleoli, and finely granular cytoplasm, arranged in nests and cords resembling decidua, accompanied by geographic necrosis—an important criterion for distinguishing them from PSN. In addition, the Ki67 proliferation index is usually greater than 10%, whereas in PSN, it is typically below 5%. Tumor cells stain positive for p63 and are negative for hPL and CD146, but this does not allow us to differentiate between a PSN because both have similar expression.

In contrast, PSTTs, the second intermediate trophoblast lesion of the tube, exhibit a predominantly infiltrative pattern, often involving vascular invasion, where tumor cells replace the walls of myometrial vessels. Hyaline material deposition [16] in the vessel walls is common. The cells have single nuclei, are hyperchromatic, and have abundant amphophilic cytoplasm. p63 immunostaining is negative, while hPL and CD146 are positive, with a Ki67 ranging from 10% to 30%.

Atypical PSNs are more cellular than classic PSNs, with infiltrative borders and increased nuclear atypia. Additionally, the Ki67 index is between 5% and 10%. Regarding immunohistochemical markers, their expression is similar to that in the PSN. The interpretation of Ki67 is of great diagnostic importance in this case.

In our case, the typical morphology, absence of atypia and mitosis with no necrosis, positivity for p63 with a very low Ki67, and the clinical context of an ectopic pregnancy led to the diagnosis of a tubal PSN.

## 4. Conclusion

We report an unexpected case of a PSN discovered in a fallopian tube for sterilization. Accurate pathological diagnosis is crucial to distinguish this lesion from malignant tumors such as ETT, PSTT, or squamous cell carcinoma, given the markedly different clinical implications.

PSNs are benign lesions with no metastatic potential and no known risk of recurrence. Although some authors have suggested that PSNs could be precursor lesions to various trophoblastic tumors, there is currently no evidence to support this hypothesis. As such, no specific follow-up is required after the diagnosis of a PSN.

## Data Availability

Data sharing is not applicable to this article as no datasets were generated or analyzed during the current study.
